# 토픽 모델링을 활용한 광범위 선천성 대사이상 신생아 선별검사 관련 온라인 육아 커뮤니티 게시 글 분석: 계량적 내용분석 연구

**DOI:** 10.4069/kjwhn.2023.02.21

**Published:** 2023-03-31

**Authors:** Myeong Seon Lee, Hyun-Sook Chung, Jin Sun Kim

**Affiliations:** 1 Department of Nursing, Nambu University, Gwangju, Korea; 1 남부대학교 간호학과; 2 Department of Computer Engineering, Chosun University, Gwangju, Korea; 2 조선대학교 컴퓨터공학과; 3 Department of Nursing, Chosun University, Gwangju, Korea; 3 조선대학교 간호학과

**Keywords:** Information, Inborn metabolism errors, Mothers, Neonatal screening, 정보, 선천성 대사이상, 어머니, 신생아선별검사

## Abstract

**Purpose:**

As more newborns have received expanded newborn screening (NBS) for metabolic disorders, the overall number of false-positive results has increased. The purpose of this study was to explore the psychological impacts experienced by mothers related to the NBS process.

**Methods:**

An online parenting community in Korea was selected, and questions regarding NBS were collected using web crawling for the period from October 2018 to August 2021. In total, 634 posts were analyzed. The collected unstructured text data were preprocessed, and keyword analysis, topic modeling, and visualization were performed.

**Results:**

Of 1,057 words extracted from posts, the top keyword based on ‘term frequency-inverse document frequency’ values was “hypothyroidism,” followed by “discharge,” “close examination,” “thyroid-stimulating hormone levels,” and “jaundice.” The top keyword based on the simple frequency of appearance was “XXX hospital,” followed by “close examination,” “discharge,” “breastfeeding,” “hypothyroidism,” and “professor.” As a result of LDA topic modeling, posts related to inborn errors of metabolism (IEMs) were classified into four main themes: “confirmatory tests of IEMs,” “mother and newborn with thyroid function problems,” “retests of IEMs,” and “feeding related to IEMs.” Mothers experienced substantial frustration, stress, and anxiety when they received positive NBS results.

**Conclusion:**

The online parenting community played an important role in acquiring and sharing information, as well as psychological support related to NBS in newborn mothers. Nurses can use this study’s findings to develop timely and evidence-based information for parents whose children receive positive NBS results to reduce the negative psychological impact.

## Introduction

선천성 대사이상 질환의 특징은 신생아 시기에는 증상이 잘 나타나지 않는다는 것으로, 증상이 나타난 이후에는 치료를 하더라도 이미 손상을 받은 세포가 회복되지 않아 장애아로 살거나 사망하게 된다. 그러나 조기 발견하여 특수 식이요법이나 적절한 치료를 하는 경우 정신지체 등과 같은 심각한 합병증을 예방할 수 있다[1,2]. 선천성 대사이상 신생아 선별검사는 질환을 조기에 발견해서 영유아 건강증진을 도모하고 정신지체아 발생을 예방하기 위한 목적으로 많은 나라에서 시행하고 있다[1,3]. 국내에는 1991년부터 정부 모자보건사업의 일환으로 선천성 대사이상 신생아 선별검사에 대한 지원이 시작되었고, 기존의 페닐케톤뇨증, 선천성 갑상선 기능저하증에 2006년부터 단풍당뇨증, 호모시스틴뇨증, 갈락토스혈증 및 선천성 부신 과형성증의 4개 질환을 추가, 총 6개 질환으로 지원을 확대하여 모자보건 향상에 기여했다[2,4].

광범위 선천성 대사이상 신생아 선별검사는 신생아의 발뒤꿈치에서 채취한 혈액을 이용하여 이중 질량분석기로 검사를 시행하며, 50여 종의 대사질환을 한 번에 검사할 수 있는 최신의 검사이다[1]. 국내에서는 2000년부터 2017년까지 18년간 이 검사의 효과를 평가한 결과[5] 국가적 차원에서 포괄적인 관리가 필요하다는 결론에 도달하여, 2018년 10월부터 모든 신생아에게 건강보험 급여를 적용하고 있다. 그러나 검사 항목이 많아지고 급여화로 검사 대상자 수가 증가하면서 위양성률이 증가하였다[1]. 선행연구에 따르면 이중 질량분석기 검사의 위양성률은 0.33%에서 6.0%이고[4], 재검률 또한 총검사의 5.6%로 보고되고 있다[6].

일반적으로 처음 실시한 검사 결과가 양성이거나 유효하지 않을 경우 재검사를 실시하고 재검사에서도 양성이 나올 경우 정밀검사를 포함한 확진검사를 실시한다. 초기 양성 결과는 의심 질환을 조기에 발견할 수 있다는 장점이 있지만, 침습적 검사를 다시 해야 하는 불편함이 있고 불필요한 입원이나 의료비용이 발생하기도 한다. 또한 검사 결과에 대한 정보나 의료진의 설명이 부족한 경우 부모는 불안감, 스트레스, 우울감, 수면 이상, 수유 문제 등을 경험하기도 한다[7,8]. 특히 재검사 결과가 불확실한 경우 반복적으로 검사를 하기도 하며, 확진검사를 통해 최종적으로 음성이 확인되고 자녀가 정상적으로 성장함에도 불구하고 부모들은 자녀의 잠재적인 질환이나 발달지연 등에 대해 지속적으로 걱정을 하고 두려움을 경험하기도 하는 것으로 나타났다[8,9]. 따라서 이 검사의 긍정적인 효과뿐 아니라 위양성 결과가 모자건강에 미치는 부정적인 영향에도 관심이 필요하다[1,10]. 국내외에서 위양성률을 줄이기 위한 다양한 노력을 하고 있지만, 부모들은 여전히 이 검사와 관련하여 부정적인 영향을 경험하는 것으로 보고되고 있다[7,11].

선별검사에 대한 재검사가 필요한 경우 보통 부모들은 문자 또는 전화 통보를 받게 되는데 항상 정확하고 완벽한 정보를 제공받지는 못하며, 대부분 흔한 질환이 아니고 질환명이 어려워서 알려주더라도 기억을 못하는 경우도 많다. 재검사 통보에 부모들은 당황하게 되고 인터넷 사이트를 통해 관련 정보를 검색하거나 육아 관련 온라인 육아 커뮤니티에서 비슷한 문제를 경험하고 있거나 경험했던 어머니들을 통해 정보를 획득하고 공유하며 정서적인 지지를 받기도 한다[8,12]. 특히 검사 결과를 통보받는 시점, 재검사 및 확진검사가 이루어지는 시기가 어머니들의 산후조리 기간이거나 아기가 어려 외출에 제한을 받는 시기이므로 시간 및 공간적인 제약이 없는 온라인 육아 커뮤니티는 이 검사와 관련하여 중요한 역할을 하는 것으로 보고되고 있다[13].

주산기 여성을 대상으로 간호를 제공하는 간호사는 신생아 선별검사의 시행과 관련 검사 과정을 촉진하고 부모 교육이나 추적 검사를 도울 책임이 있으며, 또한 심리적 지지를 위한 역할을 해야 한다[9,14]. 최근 이 검사와 관련하여 의료인 또는 부모의 지식과 태도를 조사한 연구[15,16], 위양성 결과의 영향과 관련한 요인 조사[1,17], 위양성 결과가 부모에게 미치는 영향에 대한 연구[7,8], 위양성 감소를 위한 전략 개발 연구[11]가 이루어지는 등 관련 주제에 대한 관심이 증가하고 있다. 그러나 국내에서는 선천성 대사이상으로 진단된 아동의 추적 관찰[5], 양성 결과 빈도와 소환율[6], 위양성률[4], 위양성 결과에 영향을 미치는 요인[1]을 조사한 연구는 있지만, 안전하고 효과적인 검사의 수행, 검사 관련 교육, 결과 통보, 재검사 및 확진검사 과정에서 간호사의 역할과 책임이 중요함에도 불구하고[14,17], 부모들의 심리적 경험 및 그들이 이 과정에서 무엇을 궁금해 하고 어떤 정보를 추구하는지에 대한 간호학적 관점에서의 국내 연구는 찾아볼 수 없었다.

온라인 육아 커뮤니티 이용이 활발해지면서 온라인 상에서 실시간으로 생성되는 비정형 데이터가 증가하고 있으며 이 안에서 어머니들이 사용하는 글과 단어는 정보의 전달과 더불어 그들의 사회‧심리적 측면을 반영하므로, 보건의료 제공자는 이를 통해 대상자의 관심사 및 정보 요구 정도를 파악할 수 있으며, 이러한 자료는 건강관리 영역에서 이들을 돕기 위한 자료로 활용할 수 있다[18,19]. 구조화된 설문지를 이용하여 자료를 수집하는 경우 자료 수집 및 분석에 시간이 소요되고 대상자들의 다양한 의견을 반영하는 데 제한이 있는 반면, 온라인 커뮤니티의 게시 글은 시간과 공간 제약이 없으며 대중의 살아있는 경험과 의견을 비정형화된 형태로 자유롭게 보여준다는 장점이 있다[20,21].

온라인 게시 글과 같은 자연어로 작성된 비구조적(unstructured) 데이터를 대상으로 정보를 추출하는 기술을 텍스트 마이닝(text mining)이라고 한다. 토픽 모델링은 텍스트 마이닝 기법을 활용한 확률 모델 알고리즘으로, 구조화되지 않은 대량의 텍스트에서 토픽과 키워드를 찾아내고 잠재되어 있는 주제들을 군집하고 분석한 결과를 시각화하여 핵심 주제를 파악할 수 있다[22]. 또한 토픽 모델링은 연관 관계가 높은 단어들로 구성된 벡터 형태의 패턴을 통해 단어와 문서의 분포를 추정하여 해당 문서의 주요 단어와 분류를 모델링하므로[23] 객관적인 분석이 가능하다는 장점이 있다. 따라서 토픽 모델링은 온라인 육아 커뮤니티 게시 글과 같이 비정형화된 텍스트 데이터로부터 의미 있는 정보와 구조를 도출해 낼 수 있는 방법으로 유용하게 활용할 수 있을 것이다.

본 연구의 목적은 온라인 육아 커뮤니티의 선천성 대사이상 신생아 선별검사와 관련된 게시 글을 토픽 모델링 기법을 이용하여 내용 분석을 함으로써 선천성 대사이상 신생아 선별검사, 검사 결과의 통보, 재검사 및 확진검사를 통해 최종 결과가 나오기까지의 어머니들의 경험과 정보 요구를 파악하기 위함이다. 본 연구의 결과는 선천성 대사이상 신생아 선별검사와 관련하여 안전하고 효과적인 간호를 수행하고, 모자건강에의 부정적인 영향을 최소화하기 위한 간호중재 개발의 기초 자료로 활용할 수 있을 것이다.

## Methods

Ethics statement: Obtaining informed consent was exempted by the Institutional Review Board of Chosun University (2-1041055-AB-N-01-2021-55) as this study used web crawling of existing data of an online community after anonymization.

### 연구 설계

본 연구는 토픽 모델링을 활용하여 선천성 대사이상 신생아 선별검사와 관련된 국내 온라인 육아 커뮤니티 게시 글의 주요 키워드와 토픽을 파악하기 위한 계량적 내용분석 연구이다.

### 연구 대상

본 연구의 대상은 국내 최대 규모의 임신·출산·육아 대표 커뮤니티인 ‘맘스홀릭 베이비 카페(https://cafe.naver.com/imsanbu)’ 게시판의 광범위 신생아 선천성 대사이상과 관련된 게시 글 들이다. 맘스홀릭 베이비 카페는 가입자가 300만 명 이상, 일일 방문자가 80만 명에 이르며 회원으로 가입한 이용자만이 온라인 커뮤니티의 게시 글을 열람 및 작성할 수 있다. 특히 커뮤니티 게시판 중 ‘육아 질문방’ 게시판은 어머니들이 개인적인 경험을 게시하고, 읽고, 답글을 작성하여 공유하는 기능의 메뉴이므로 신생아 어머니의 선천성 대사이상 검사 관련 정보 요구를 파악하기 위해 적합한 것으로 판단되어 해당 카페 운영자에게 연구에 대해 고지 후 해당 게시 글을 활용하였으며 게시 글 작성자와 관련된 자료는 자료 수집에서 제외하였다.

### 자료 수집

파이썬(Python) 프로그래밍 언어로 웹 크롤러(web crawler)를 구현하여 데이터를 수집하였다. 온라인 커뮤니티 게시 글의 동적 크롤링을 위해 셀레니움 라이브러리(Selenium Library)를 사용하였으며 크롬 웹드라이버(Chrome WebDriver)로 생성되는 가상 브라우저와의 연동을 통해 게시 글로부터 필요한 데이터만 추출하도록 프로그래밍하였다. 자료 수집을 위한 키워드는 ‘선천성대사이상검사’, ‘신생아선천성대사이상검사’, ‘신생아대사이상검사’로 지정하였다. 광범위 선천성 대사이상 신생아 선별검사가 2018년 10월부터 건강보험의 급여 항목으로 적용된 부분을 고려하여 2018년 10월 1일부터 2021년 8월 31일까지 올려진 게시 글을 게시물 작성자의 아이디를 제외하고 수집하였으며 글의 제목, 작성 일자, 본문 내용, 댓글을 하나의 행으로 하는 ‘CSV’ 형식의 파일로 저장하였다. 크롤링 된 자료에서 중복된 데이터는 삭제하여 총 1,127건의 게시 글을 1차로 저장하였다. 1차로 저장된 자료에서 분석에 포함할 자료를 선정하기 위해 제외 기준을 마련하였고, 연구자 간의 반복적인 4회차의 분석 회의를 통해 연구자의 주관성을 배제하고 게시 글 선정에 신뢰성을 확보하였다. 연구자가 각자 게시 글의 본문을 검토한 뒤 선천성 대사이상 검사에 대한 구체적인 내용이 아닌 단순한 검사 여부에 대한 글, 조리원 후기에 대한 글 등 연구의 목적에 부합하지 않은 것으로 판단되는 경우 분석 대상에서 제외하고 최종 634건의 게시 글을 선정하였다.

### 자료 분석

선정된 게시 글을 대상으로 단어 추출 및 정제, 키워드 분석, 토픽 모델링 분석을 실시하였다. 본 연구자는 연구에 사용된 통계프로그램인 NetMiner 4.4 프로그램을 개발한 ㈜사이람(Cyram, Seongnam, Korea)의 텍스트 네트워크 및 토픽 모델링에 대한 통계 분석 관련 교육을 수강하였으며, 연구 과정 중 지속적으로 ㈜사이람의 교육팀에 자문을 구하였다.

#### 단어 추출 및 정제

자료 분석 대상으로 선정된 게시 글은 개별 인식번호, 작성일자, 제목, 본문, 댓글로 구성된 MS Office 엑셀 파일(Excel, Microsoft, Redmond, WA, USA)로 변환하였다. 그 후 NetMiner 4.4 프로그램[24]에서 제공하는 자연어 처리 과정을 거쳐 단어를 추출하였다. 자연어 처리 과정에서 대명사, 숫자, 부사 같은 불용어(stopword)는 자동으로 제외되고 주요 개념을 파악하기 위해 추출 단어의 품사는 ‘명사(noun)’로 지정하고, 연구자가 등록한 지정어(defined words), 유의어(thesaurus), 제외어(exception list) 사전을 적용하여 의미형태소를 추출하였다.

본 연구에서 단어 정제를 위한 지정어, 유의어, 제외어 사전의 개발 과정은 다음과 같다. 2인의 연구자가 사전을 적용하지 않고 추출된 전체 단어 목록을 살피면서 작업을 진행하였다. 지정어 사전은 NetMiner 프로그램 분석 상 한 개의 형태소를 기본 단위로 인식하기 때문에, 두 개 이상의 형태소로 구성된 고유명사나 복합명사들은 지정어 사전을 통해 한 개의 단어로 인식하도록 설정하였다. ‘선천성갑상선기능저하증’, ‘아미노산대사장애’, ‘선천성대사이상검사’ 등의 단어들이 분리되지 않도록 지정어로 등록하였다. 유의어 사전은 동일하고 비슷한 의미를 가지나 표기가 다른 단어와 띄어쓰기가 다르게 쓰인 동일어를 모아 하나의 대표어로 등록하는 사전이다. 선천성 대사이상 질환명의 경우 「제8차 한국표준질병ㆍ사인분류」[25]를 참고하여 대표어를 등록하였다. 예를 들어, ‘티로신혈증’, ‘타이로신3형’은 ‘타이로신혈증’으로 등록하였다. 제외어 사전은 일반적인 개념을 나타내는 단어나 분석에서 제외할 단어를 지정하는 사전으로, 게시 글에 많이 사용되는 ‘필독’, ‘맘님’, ‘그동안’, ‘그때’, ‘오랜만’, ‘나날’ 등과 지역명을 제외어로 등록하였다. 또한 의미를 파악하기 어려운 한 음절 글자는 Netminer의 Query 기능을 이용하여 모두 삭제하여 두 음절 이상의 형태소만 분석에 포함하였다. 이 과정에서 연구자들이 동의하지 않는 경우, 게시 글 본문을 다시 읽어보고 단어가 사용된 맥락을 확인하는 검토 및 논의를 통하여 최종 결정하였다.

#### 키워드 분석

전체 게시 글의 주요 속성을 파악하기 위하여 단어빈도(term frequency, TF)와 단어빈도-역문서 빈도(term frequency-inverse document frequency, TF-IDF) 분석을 수행하였다. 출현 빈도 값으로 자주 등장하는 단어를 확인할 수 있지만, “선천성대사이상검사”와 같이 빈도 값이 지나치게 큰 키워드는 검색어 자체이므로 출현 빈도 값이 높다고 하여 중요한 키워드라고 볼 수 없다. TF-IDF는 TF와 IDF의 곱으로, TF는 특정 문서 하나에서 특정 단어가 나온 횟수이며, IDF는 특정 단어의 문서 내 빈도를 역수로 취한 값이다. 따라서 TF-IDF 값은 특정 문서에서 단어 빈도가 높을수록, 그리고 전체 문서들 중 그 단어를 포함한 문서가 적을수록 높아져서 어떤 단어가 특정 문서에서 얼마나 중요한 것인지를 나타내므로, 일반적인 개념을 제외하고 유의미한 단어를 추출하는 데 사용된다[26].

본 연구에서는 TF-IDF 가중치를 적용하여 키워드를 추출하였다. 선행연구[26]를 참조하고 TF-IDF 값에 따라 삭제되는 단어들을 검토한 후 TF-IDF 값을 0.5 이상으로 설정하여 키워드를 필터링하였으며, 출현 빈도와 TF-IDF 값을 기준으로 상위 키워드 30개를 각각 추출하였다.

#### 토픽 모델링 분석

토픽 모델링은 문서의 주제, 즉 토픽을 도출하기 위해 텍스트 내 단어를 분석하는 방법으로[22], 본 연구에서는 가장 일반적으로 사용되고 있는 잠재 디리클레 할당(latent Dirichlet allocation, LDA) 알고리즘을 사용하였다. LDA는 문서가 여러 개의 토픽으로 구성되며 토픽은 키워드의 집합이라고 가정하여 문서와 단어로 구성된 행렬을 기반으로 토픽을 확률적으로 추론해내는 방법이다[27]. 즉 온라인 게시 글과 댓글들을 구성하는 키워드 사이에는 잠재된 토픽이 있으며 토픽은 전체 게시 글과 댓글의 주제범주를 나타내 준다고 말할 수 있다. 토픽 수 및 LDA 파라미터(parameter)가 이용되며 분석 시 연구자가 입력해야 한다. 본 연구에서는 LDA 파라미터를 선행연구[28]에 근거하여 사전 확률분포 α를 0.1, 토픽 내 사전확률분포 β를 0.01, 반복 수행 횟수를 1,000회로 설정하여 실시하였다. 토픽의 수는 통계적인 방법과 해석적인 방법을 사용하여 결정하였다. 통계적인 방법은 K-means clustering을 이용한 실루엣 계수(silhouette coefficient) 값을 참고하였다. 실루엣 계수 값은 각 데이터가 얼마나 조밀하게 모여 있는지를 나타내는 값으로, 1에 가까울수록 군집들이 적절히 분리되었다고 볼 수 있다[29]. 해석적인 방법으로는 주제 범주화가 잘 되었다고 판단되는 토픽 수로 선택하라는 권고에 따라[27], 유효한 실루엣 계수로 나타난 토픽 수를 바꾸어 넣어가며 한 개의 토픽으로 묶인 게시 글의 주제를 살펴보고 연구팀의 협의를 통해 최종 결정하였다. LDA 분석 결과로 나타난 토픽 그룹의 이름은 토픽 내 가중치가 높은 키워드 10–20개와 토픽 확률이 높은 상위 게시 글의 본문 내용을 참고하여 연구자들이 논의 후 명명하였다. 토픽 내 가중치를 적용한 각 토픽별 워드 클라우드를 도출하였으며 가중치가 높은 10개의 키워드는 토픽-단어 네트워크를 이용하여 토픽과 키워드의 관계를 스프링 맵(spring map)으로 시각화하였다.

## Results

### 신생아 선천성 대사이상 관련 게시 글의 키워드

**Figure 1.**
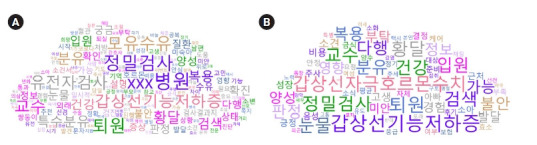
Visualization of the top 500 keywords by term frequency (A) and term frequency-inverse document frequency (B).

총 634건의 게시 글에서 1,057개 단어가 추출되었다. 이들 중, 출현빈도가 상위 30위인 단어들을 살펴보면 ‘XXX 병원’이 231건으로 가장 많았으며, ‘정밀검사’ 215건, ‘퇴원’ 205건, ‘모유수유’ 196건, ‘갑상선기능저하증’ 191건, ‘교수’ 189건, ‘유전자검사’, ‘분유’ 161건, ‘황달’ 158건, ‘특수분유’ 151건 등의 순이었다. 또한 TF-IDF로 본 중요도가 높은 상위 키워드를 살펴보면 ‘갑상선기능저하증’이 가장 높았고, ‘퇴원’, ‘정밀검사’, ‘갑상선자극호르몬수치’, ‘황달’, ‘교수’, ‘검색’, ‘건강’, ‘눈물’, ‘입원’ 등의 순이었다(Table 1). 출현 빈도와 TF-IDF 값을 기준으로 상위 500개의 키워드를 워드 클라우드로 각각 시각화하였다(Figure 1).

### 신생아 선천성 대사이상 관련 게시 글의 토픽 모델링

#### 토픽 수 설정

**Figure 2.**
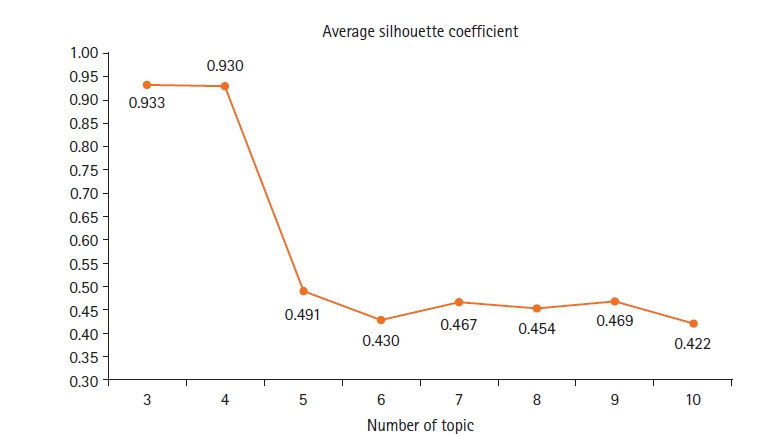
Silhouette coefficient to the number of topics.

실루엣 계수 산출 결과, 실루엣 계수 값이 높게 나타나는 토픽 수는 3개(.933)와 4개(.930)였다(Figure 2). 이에 연구팀은 3개와 4개로 토픽의 수를 변경해가면서 토픽 수의 타당성을 검토한 결과, 주제 범주화가 잘 되어 광범위 신생아 선천성 대사이상 검사와 관련하여 해석적인 적합성이 높은 4개로 판정하였다.

#### 토픽 모델링

선천성 대사이상 신생아 선별검사와 관련한 게시 글들에 잠재된 토픽 수를 4개로 설정하여 LDA를 활용한 토픽 모델링 분석 후 먼저 4개의 토픽의 주요 키워드와 확률을 확인하였다. 토픽 1과 토픽 3은 ‘퇴원’이, 토픽 2와 토픽 4는 ‘교수’가 공통의 핵심 키워드로 나타났다. 토픽의 주요 키워드와 토픽 해당 확률이 높은 게시 글의 본문을 확인하여 토픽명을 다음과 같이 명명하였다(Table 2).

토픽 1은 전체 토픽의 21.6%로 네 개의 토픽 중 가장 적은 비중을 차지하고 있었으며, 주요 키워드는 모유수유, 분유, 황달, 퇴원, 수유, 검사결과지, 미숙아, 신생아 집중치료실, 문자, 입원 등이었다. 할당 확률이 높았던 대표적인 게시 글을 분석한 결과, 선천성 대사이상과 황달과의 연관성, 선천성 대사이상 선별검사에서 이상 수치를 보인 경우 재검사 전에 모유수유를 지속해도 될지에 대한 부분과 분유 수유에 대한 경험과 정보를 요구하는 내용으로 이루어져, 토픽 1은 ‘선천성 대사이상 관련 수유’로 명명하였다.

토픽 2는 전체 토픽의 26.5%의 비중을 차지하고 있었으며, 주요 키워드는 갑상선 기능 저하증, 복용, 갑상선 자극 호르몬 수치, 신지로이드, 교수, 갑상선 자극 호르몬, 호르몬, 영향, 발달, 갑상선 기능 항진증 등이었다. 할당 확률이 높았던 게시 글을 분석한 결과, 산모가 갑상선 기능 저하증 또는 항진증이거나 신생아가 대사이상 검사에서 갑상선 자극 호르몬 수치에 이상이 있는 경우에 추가적인 갑상선 자극 호르몬 검사와 관련된 경험과 갑상선 기능 이상이 신생아의 발달에 미치는 영향 등에 대한 정보를 요구하는 내용으로 이루어져, 토픽 2는 ‘갑상선 기능 문제를 가진 산모와 신생아’로 명명하였다.

토픽 3은 전체 토픽의 22.0%의 비중을 차지하고 있었으며, 주요 키워드는 퇴원, 설명, 검진, 검색, 남편, 외래, 발뒤꿈치 채혈, 산부인과, 눈물, 코로나 등이었다. 할당 확률이 높았던 게시 글을 분석한 결과, 분만 후 퇴원한 산모가 산후조리 중에 선천성 대사이상 재검사와 관련된 연락을 받고 외래를 통한 재검사 절차, 채혈 방법, 코로나 상황에서 병원 진료 등에 대한 경험과 정보를 요구하는 내용으로 이루어져, 토픽 3은 ‘선천성 대사이상 선별검사 재검사’로 명명하였다.

토픽 4는 전체 토픽의 29.0%로 가장 높은 비중을 차지하고 있으며 주요 키워드로는 XXX 병원, 유전자 검사, 정밀검사, 특수 분유, 양성, 교수, 확진, 지방산 대사장애, 아미노산 대사장애, 질환 등이었다. 할당 확률이 높았던 게시 글을 분석한 결과, 신생아 선천성 대사이상 선별검사의 재검사에서도 이상 수치를 보인 경우, XXX 병원 및 3차 종합병원 이상에서 가능한 정밀검사 및 유전자 검사와 확진을 받는 과정에 대한 경험과 정보를 요구하는 내용으로 이루어져, 토픽 4는 ‘선천성 대사이상 확진검사’로 명명하였다.

**Figure 3.**
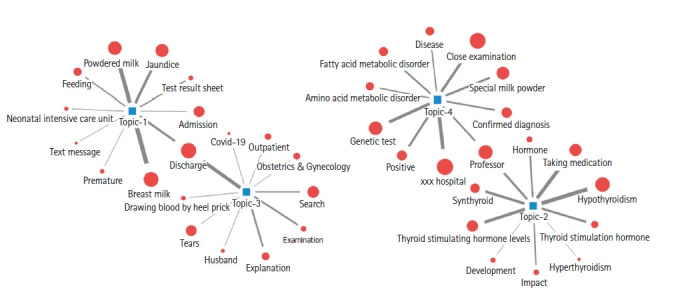
Topic network of the main keywords.

**Figure 4.**
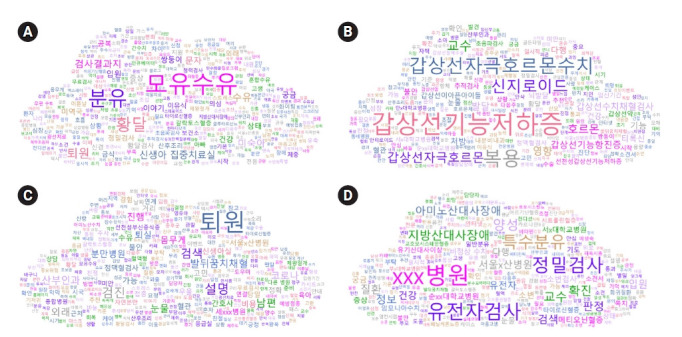
Word cloud of topic keywords. (A) Topic 1: feeding related to IEMs. (B) Topic 2: mother and newborn with thyroid function problems. (C) Topic 3: retests of IEMs. (D) Topic 4: confirmatory tests of IEMs. IEM: Inborn error of metabolism.

토픽 4개와 토픽의 상위 확률 분포를 차지하는 주요 키워드 10개의 네트워크를 스프링 맵을 통해 시각화하였고(Figure 3), 각 토픽의 주요 키워드의 워드 클라우드는 Figure 4와 같다.

## Discussion

신생아 선천성 대사이상 선별검사와 관련하여 온라인 육아 커뮤니티 게시 글로부터 도출된 4개의 토픽과 주요 키워드를 중심으로 논의해 보고자 한다. 토픽이 차지하는 비중 순서로 본 논의를 진행하였다.

토픽 4 ‘선천성 대사이상 확진검사’는 전체 토픽 중 가장 큰 비중을 차지하였다. 이 토픽에서 가장 영향력이 큰 키워드는 ‘XXX 병원’이었는데, 이 키워드는 온라인 게시 글에 출현 빈도도 가장 높았다. 재검사에서도 양성이 나오는 경우 확진을 위한 정밀검사를 하는데, 현재 국내에서 확진검사가 가능한 병원은 11개이다. 이들 병원 중 XXX 병원을 제외하고는 모두 대학병원으로 예약, 검사, 결과 통보까지 수주에서 수개월이 걸린다. 반면 유전학 연구소를 직접 운영하고 있는 XXX 병원은 대학병원과는 다르게 예약 대기가 짧고 확진검사 후 24–48시간 내에 결과를 확인할 수 있는 장점이 있어 부모들이 선호하는 병원이다. 토픽 4의 또 다른 핵심 키워드는 ‘정밀검사’였다. 어머니들은 퇴원 상태에서 진행되는 정밀검사를 위한 병원 선택, 예약, 검사 진행 및 결과 확인 등에 대한 정보 요구도가 높고 정보검색 과정에서 어려움을 경험하고 있었다. 외국의 선행연구에서도 가족에게 정밀검사를 위한 예약의 어려움, 검사 후 최종 결과가 나오기까지 기다리는 시간이 큰 고통인 것으로 보고하고 있다[8,12]. 따라서 이런 부모들의 심리적 요인을 고려하여 재검사, 확진검사 과정을 보다 체계화, 신속화하기 위한 노력이 요구된다[30,31].

토픽 2 ‘갑상선 기능 문제를 가진 산모와 신생아’는 4개의 핵심 주제 중 비중이 두 번째였다. ‘갑상선 기능 저하증”은 출현 빈도 상위 5위였으며, TF-IDF 값 기준으로 중요도가 가장 높았던 키워드였다. 산모의 갑상선 질환력 및 갑상선약 복용력[2], 미숙아[30,32], 황달[2]등 다양한 주산기 요인이 갑상선 호르몬 수치에 영향을 미치므로 재검사 및 여러 차례 추가검사를 요하는 경향이 있기 때문에 관심과 관련 정보 요구가 많았을 것으로 추정한다. 선천성 갑상선 기능 저하증은 선천성 대사이상 검사 항목 중 가장 발생빈도가 높은 내분비계 질환[5,33]이다. 선천성 갑상선 기능 저하증은 출생 초기에 발견해 호르몬을 보충해주면 괜찮지만 치료 시기가 늦어지면 정신지체로 이어질 수 있어 조기 발견 및 조기 중재가 중요한 질환이다[3]. 이러한 점을 고려할 때 주산기 여성을 대상으로 특히 갑상선 기능 및 태아 및 신생아에 미치는 영향, 갑상선 기능 검사 등과 관련된 교육이 강화되어야 할 것이다.

토픽 3은 ‘선천성 대사이상 선별검사 재검사’로 4개의 핵심 주제 중 비중이 세 번째였고, 이 토픽의 핵심 키워드는 ‘퇴원’이었다. 외국의 선행연구를 보면 퇴원 후 선별검사에서 양성 통보를 받은 부모들은 정보를 얻기 위해 인터넷 검색을 하지만 관련 정보는 매우 제한적이며, 검색하면서 질환의 경과 및 예후 등에 대한 무서운 정보를 접하면서 두려움, 불안 등을 경험하고 오히려 고통이 가중되는 것으로 나타났다[8]. 따라서 퇴원 교육에 본 검사 관련 정보지 제공이나 근거 중심의 정보를 얻을 수 있는 웹 사이트, 상담 가능자의 연락처 등 구체적인 정보 제공이 필요하다. 선별검사 양성 결과는 혈액 채취 시 아기가 검사에 적당한 조건을 만족하지 않아 양성으로 나오는 경우, 즉 위양성인 경우가 대부분임에도 불구하고 해당 검사 결과 및 재검사에 대한 의료진의 자세한 설명이나 정보 제공이 부족한 경우 어머니는 당황하고 심리적인 부담감 및 걱정, 두려움, 스트레스 등 부정적인 경험을 하며 산후조리나 육아에 집중을 하지 못하고[1,34], 아기의 건강과 발달에 대해 장기적인 걱정과 두려움을 겪는 것으로 보고되고 있다[35]. 따라서 산부인과/신생아실 간호사를 포함한 의료진들은 위양성 결과의 발생 빈도를 줄이고, 검사와 관련하여 신생아 및 부모에게 발생할 수 있는 부정적 영향을 최소화하기 위해 다음과 같은 노력이 필요하다[1,10]. 첫째, 신생아실 및 신생아 중환자실 간호사를 포함한 의료진은 검사물 채혈 시기 및 방법에 있어서 세심한 주의가 필요하다. 선별검사는 신생아의 수유 상태가 좋은 경우 출생 후 3–7일에 시행할 것을 권장하지만, 수유 상태가 좋지 않은 미숙아 또는 저출생 체중아는 5–7일에 시행하고 수유 상태가 좋아지면 다시(생후 2주경) 하도록 하고 있다. 또한 정확한 검채물을 위해 종이 필터의 유효기간 체크, 적절한 양의 샘플링 등 검채 수집에도 주의를 기울여야 한다. 실제로 부적절한 검채 시기 및 검채물은 위양성 증가의 한 원인이며[1,10] 미숙아나 저체중아에게 위양성률이 높은 것으로 보고되고 있다[36]. 이를 위해 외국의 선행연구에서는 신생아실, 신생아 중환자실 간호사를 포함한 의료진 교육을 강화하고 엄격한 검사 프로토콜을 개발 및 적용할 것을 권장하고 있다[36,37]. 둘째, 산부인과/신생아실 간호사는 선별검사에 대한 구두 및 서면 정보지 제공 시 재검 가능성 및 재검사의 의미에 대한 정보를 직접적으로 제공할 뿐만 아니라, 구두 및 서면 정보지에 관련 정보를 제공하는 사이트에 대한 정보를 포함하도록 한다. 선천성 대사이상 신생아 선별검사 관련 유럽의 26개국에서 부모에게 제공하는 정보지 내용을 분석한 한 연구[38]에서 주장한 것처럼 정보지에 가능성이 낮지만 위양성이 나올 수 있고 그 경우 불가피하게 의심이 되는 질환이 아니라는 것을 입증하기 위해 추가 검사가 필요하다는 정보를 포함하고, 산부인과/신생아실 간호사는 부모 교육에 현재 제공되고 있는 정보에 추가하여 위양성 결과의 빈도, 선천성 대사질환의 증상, 구체적인 추후검사 과정 등의 정보를 제공할 것을 권장한다. 또한 양성 통보를 받았을 때 온라인 상에서 찾아볼 수 있는 정보가 제한적이었다는 대상자들의 고충을 고려할 때 산부인과/신생아실 간호사는 정보지나 온라인 커뮤니티에 사용자가 좀 더 편리하게 찾아볼 수 있는 웹 사이트 링크를 제공해 주는 방법도 고려할 수 있다[8,38]. 또한 이러한 정보 제공지나 웹 사이트의 정보는 부모들이 이해 가능하고 실행 가능한 수준으로 개발되어야 한다[39]. 그리고 검사 결과는 이 검사에 대해 전문성을 가진 의사, 간호사, 또는 임상유전 상담가가 통보하여 부모가 필요한 질문이나 상담을 할 수 있도록 할 것을 권장한다[8,38]. 셋째, 산부인과/신생아실 간호사는 이 검사와 관련된 충분한 지식과 부모 상담 및 지지 역량이 필요하다. 많은 산모들이 출산 후 간호사로부터 선별검사에 대한 정보를 접하게 되지만, 이 시기 어머니는 산후회복 및 육아 등으로 새로운 정보를 획득하는 데 신체적 및 심리적으로 제한이 있을 수 있으므로 산전교육 또한 중요하다. 의료인과 가족 간의 의사소통은 산전관리 기간부터 시작되어야 하며 산후 혈액 채취 시 추가적인 정보를 제공하는 것이 필요하다[17]. 또한 신생아실 또는 신생아 중환자실 간호사는 재검사 통보, 질환, 확진검사에 대한 설명 등 이 검사와 관련된 과정에서 가족 교육, 상담 및 추적 조사를 도와줄 책임이 있으며 이 과정에서 가족의 지지를 위해 중요한 역할을 해야 한다[10]. 따라서 산부인과/신생아실 간호사는 이 검사 및 질환에 대한 충분한 지식과 기술이 있어야 하며[34] 또한 대사이상 질환들의 유전적 특징 때문에 유전학에 대한 역량을 갖추어야 한다[14,17]. 넷째, 선별검사에서 양성 판정을 받은 경우 그것이 위양성임이 밝혀질 때까지 불확실성 속에서 부모가 겪는 스트레스와 걱정 등의 경감을 위한 의료진의 의사소통 역량은 매우 중요하다[4,37]. 그러나 외국의 선행연구는 간호사를 포함한 의료진은 선별검사 및 유전질환에 대한 이해가 부족하여 관련 대화를 하는 데 자신감이 부족한 것으로 보고하고 있다[15]. 간호사와 의사를 대상으로 본 검사에 대한 지식을 조사한 한 홍콩 연구[15]에서 대상자의 47.5%가 선천성 대사질환에 대해서는 잘 알지 못했고, 73.6%는 광범위 선천성 대사이상 검사에 대해 잘 알지 못하는 것으로 나타났다. 따라서 산부인과/신생아실 간호사는 이 검사와 관련 지식과 기술을 갖추고[34], 상담 및 리스크 커뮤니케이션 역량을 갖추기 위한 교육 및 훈련이 필요할 것이다[4]. 또한 선별검사 양성 결과의 의미와 해석은 가족에게 누가 어떻게 설명하는지에 따라 다르게 받아들여질 수 있다[8]. 호주 연구진이 실시한 간호사의 유전 상담 실무 관련 통합적 고찰 연구[40]에서 간호사는 유전과 관련된 건강 문제를 예방하고 유전 문제를 가진 가족을 지지하는 중요한 역할을 할 수 있으며, 간호사가 제공한 유전 상담에 만족도가 높은 것으로 나타났다. 국내에서는 간호사가 임상에서 유전 상담 간호사로서 역할을 하는 경우는 드물며, 간호 교육과정에서 매우 제한적으로 다루고 있는 실정이다. 그러나 유전 간호 실무의 역할 및 필요성이 대두되면서 간호 교육에서 유전 간호 역량을 강화시키기 위한 노력이 요구되고 있다[41,42].

토픽 1 ‘선천성 대사이상 관련 수유’는 4개의 핵심 주제 중 비중이 네 번째였고, ‘분유’와 ‘모유수유’가 이 토픽의 주요 키워드였다. 양성 결과가 나오면 재검사를 하게 되고 필요 시 모유나 일반 분유 수유를 중단하고 특수 분유를 먹이는 것 등과 관련된 정보를 추구하는 것으로 나타났다. 선천성 대사질환을 가진 신생아는 태어날 때부터 체내에 특정 효소가 없거나 부족해 일반 분유(모유)를 정상적으로 소화·흡수·분해하지 못하기 때문에 선별검사에서 양성이 나와 재검사 또는 확진검사를 하고 기다리는 동안 모유 또는 일반 분유 수유를 중단하고 질환이 아님을 확인할 때까지 특수 조제 분유를 먹여야 한다[8,43]. 확실한 진단이 나올 때까지 수주에서 수개월이 소요될 수 있으므로 산부인과/신생아실 간호사를 포함 의료진들이 이 기간 동안의 수유에 대한 적절한 정보를 제공하고 교육하는 것이 중요하다[8]. 특히 본 연구에서 대상자들은 퇴원 후 수유와 관련된 불안, 스트레스, 불확실성 등을 경험했기 때문에 간호사는 특수 분유가 필요한 선천성 대사질환을 진단받은 경우 관련 의료인들과 협력하여 대상자들의 퇴원 교육에 수유 관련 정보를 강화하고, 퇴원 후에도 수유 관련 정보를 얻을 수 있는 웹 사이트, 수유관련 상담이 필요한 경우 연결 가능한 부서나 전문 상담사의 연락처 등에 대한 구체적인 정보 제공이 필요함을 인식하고 실무에 반영하여야 할 것이다.

본 연구는 광범위 신생아 선천성 대사이상 선별검사와 관련 어머니들의 경험과 정보 요구를 신생아 어머니들의 활발한 의사소통 통로인 온라인 커뮤니티의 게시 글과 댓글을 통해 조사하였다는 점과, 토픽 모델링을 활용한 핵심 주제 범주화를 통해 해당 검사와 관련한 주산기 간호의 질을 향상하기 위한 기초 자료를 제공하고 간호사의 책임과 역할을 규명하였다는 점에서 의의가 있다. 그러나 본 연구는 국내의 한 온라인 육아 커뮤니티를 분석한 연구이기 때문에 연구 결과를 일반화하는데 신중을 기할 필요가 있다. 또한 국내 최대 규모의 사이트를 대상으로 하였으나 해당 커뮤니티는 여성 회원만이 가입할 수 있어, 아버지의 경험 및 정보 요구는 파악할 수 없었다는 제한점이 있다. 아버지도 재검사 및 확진검사 등에 대한 의사 결정에 많이 관여할 수 있는 상황이므로 아버지들이 참여할 수 있는 육아 관련 온라인 커뮤니티 운영의 필요성을 제안한다. 온라인 게시 글의 특성상 온라인 접근이 용이한 계층이 다수 포함되어 있을 가능성이 있으므로 일반화에 신중을 요한다. 또한 연구자가 사전을 통한 통제 노력을 했으나 자료 분석 과정에서 온라인 게시 글이 뉴스나 일반 문헌에 비해 축약어 등을 사용하고 있어서 형태소 분석기에 의존한 전처리 과정이 키워드를 완벽하게 추출하지 못했을 가능성이 있다.

본 연구 결과를 근거로 광범위 신생아 선천성 대사이상 검사와 관련된 간호실무, 간호연구 및 간호교육의 향상을 위해 다음과 같이 제언한다:

선천성 대사이상 신생아 선별검사 관련 온라인 육아 커뮤니티 게시 글의 주요 내용은 검사 시행 후 양성 통보를 받은 경우 재검사 또는 확진검사, 질환 관련 질문, 정보 및 경험 공유, 심리적 지지 등이 많았다. 온라인 육아 커뮤니티는 본 검사 관련 유사한 경험을 한 어머니끼리 정보 획득 및 공유뿐 아니라 공감, 위로 등의 사회‧심리적 지지 등 중요한 역할을 함을 확인할 수 있었다. 최근 온라인 커뮤니티에서 임신과 출산에 대한 여성들의 관심사[18]나 영유아 어머니의 양육 관련 질문 내용 분석 게시 글을 분석한 결과[19]에서도 나타난 것처럼, 온라인 커뮤니티는 시간과 공간의 장벽에 방해를 받지 않으므로 특히 신생아 어머니들이 자신이 원하는 시간에 손쉽게 정보를 교환하고 소통할 수 있는 좋은 장이었다. 따라서 산부인과/신생아실 간호사는 다학제적 협력을 통해 이 검사와 관련 양성 결과의 통보, 재검사 및 확진검사까지 불확실성을 경험하는 전 과정에서 가족에게 적절한 정보 제공뿐 아니라 심리적 지지 및 스트레스 감소 등을 위해 중요한 역할을 해야 할 것이다. 또한 이 검사와 관련하여 어머니들의 소통의 장인 온라인 커뮤니티에 지속적인 관심을 가지고, 대상자들의 경험과 정보 요구를 파악하여 온라인 커뮤니티를 활용한 정보 제공, 중재의 개발 및 적용을 할 필요가 있다.

외국에서는 선천성 대사이상 신생아 선별검사와 관련한 서비스의 질을 향상시키고 이 검사로 인한 부정적인 영향을 줄이고자 다양한 연구가 진행되고 있다. 이 검사와 관련된 대상자들의 경험 및 정보 요구는 각 국가의 검사 관련 의료체계 및 문화에 따라 다를 수 있으므로, 우리나라의 관련 서비스 질 향상을 위해서 부모와 의료 제공자들을 대상으로 좀 더 심도 있고 체계적인 연구가 요구된다. 부모와 의료인들을 대상으로 심층 면담 또는 초점 집단 면담을 통해 이 검사와 관련하여 장점은 극대화하고 부정적인 영향은 최소화할 수 있는 효과적인 방법을 모색하려는 지속적인 관심과 연구가 요구된다. 또한 간호사가 선천성 대사이상 신생아 선별검사 관련한 실무를 효과적으로 수행하기 위해서는 간호 교육과정에서 유전 간호 역량을 강화하기 위한 노력이 요구된다.

## Figures and Tables

**Table 1.** Top 30 keywords by frequency and TF-IDF

Rank	Frequency		TF-IDF	
	Keyword	n	Keyword	Value
1	XXX Hospital	231	Hypothyroidism	42.66
2	Close examination	215	Discharge	41.75
3	Discharge	205	Close examination	39.57
4	Breastfeeding	196	Thyroid-stimulating hormone levels	38.95
5	Hypothyroidism	191	Jaundice	38.89
6	Professor	189	Professor	38.31
7	Genetic test	185	Search	38.13
8	Powdered milk	161	Health	37.72
9	Jaundice	158	Tears	37.51
10	Special milk powder	151	Admission	36.43
11	Health	146	Powdered milk	36.25
12	Taking medication	145	Breast milk	35.85
13	Search	143	Relief	35.54
14	Thyroid-stimulating hormone levels	141	Anxiety	33.65
15	Admission	128	Taking medication	33.58
16	Tears	122	Synthroid	32.98
17	Confirmed diagnosis	120	Diagnosis	32.94
18	Positive	115	Genetic test	32.6
19	Synthroid	111	Check	31.67
20	Relief	109	Possibility	31.57
21	Diagnosis	107	Information	31.11
22	XX Medical Center	106	Obstetrics & Gynecology	30.91
23	Fatty acid metabolic disorder	105	Curiosity	30.88
24	Curiosity	102	Special milk powder	30.79
25	Disease	102	Disease	29.97
26	Anxiety	97	Confirmed diagnosis	29.53
27	Information	95	Positive	29.16
28	Check	93	XXX Hospital	29.13
29	Amino acid metabolic disorder	89	XX Medical Center	29.07
30	Feeding	88	Amino acid metabolic disorder	28.88

TF-IDF: Term frequency-inverse document frequency.

**Table 2.** Results of topic modeling

Rank	Topic 1		Topic 2		Topic 3		Topic 4	
	Feeding related to IEMs		Mother and newborn with thyroid function problems		Retests of IEMs		Confirmatory tests of IEMs	
	Main keyword	Probability	Main keyword	Probability	Main keyword	Probability	Main keyword	Probability
1	Breastfeeding	.056	Hypothyroidism	.055	Discharge	.045	XXX hospital	.045
2	Powdered milk	.046	Taking medication	.041	Explanation	.020	Genetic test	.036
3	Jaundice	.034	Thyroid-stimulating hormone levels	.040	Examination	.020	Close examination	.034
4	Discharge	.023	Synthroid	.032	Search	.018	Special milk powder	.030
5	Feeding	.018	Professor	.024	Husband	.015	Positive	.023
6	Test result sheet	.017	Thyroid-stimulating hormone	.023	Outpatient clinic	.015	Professor	.020
7	Premature	.016	Hormone	.019	Drawing blood by heel prick	.014	Confirmed diagnosis	.020
8	Neonatal intensive care unit	.016	Impact	.017	Obstetrics & gynecology	.014	Fatty acid metabolic disorder	.020
9	Text message	.014	Development	.015	Tears	.014	Amino acid metabolic disorder	.017
10	Admission	.012	Hyperthyroidism	.014	COVID-19	.013	Disease	.017
Total, n (%)	137 (21.6)		168 (26.5)		140 (22.0)		190 (29.0)	

IEM: Inborn error of metabolism.

## References

[1] Kim TK, Lee SH, Yu ST, Oh YK (2019). Analysis of major factors affecting false positive results in neonatal screening test within 3 days after birth. Perinatol.

[2] Lee DH (2014). The past, present, future of newborn screening in Korea. J Korean Soc Inherit Metab Dis.

[3] O'Connor K, Jukes T, Goobie S, DiRaimo J, Moran G, Potter BK (2018). Psychosocial impact on mothers receiving expanded newborn screening results. Eur J Hum Genet.

[4] Kim H, Shin SM, Ko SY, Lee YK, Park SW (2016). Investigation of false positive rates newborn screening using Tandem Mass Spectrometry (TMS) technology in single center. J Korean Soc Inherit Metab Dis.

[5] Song WJ, Lee S, Jeon YM, Kim SZ, Jang MY (2018). 18-year follow-up of expanded newborn screening for metabolic and endocrine disorders. J Korean Soc Inherit Metab Dis.

[6] Cho SE, Park EJ, Seo DH, Lee IB, Lee HJ, Cho DY (2015). Neonatal screening tests for inherited metabolic disorders using tandem mass spectrometry: experience of a clinical laboratory in Korea. Lab Med Online.

[7] Schmidt JL, Castellanos-Brown K, Childress S, Bonhomme N, Oktay JS, Terry SF (2012). The impact of false-positive newborn screening results on families: a qualitative study. Genet Med.

[9] DeLuca J, Zanni KL, Bonhomme N, Kemper AR (2013). Implications of newborn screening for nurses. J Nurs Scholarsh.

[10] Joseph RA (2017). Expanded newborn screening: challenges to NICU nurses. Adv Neonatal Care.

[11] Malvagia S, Forni G, Ombrone D, la Marca G (2020). Development of strategies to decrease false positive results in newborn screening. Int J Neonatal Screen.

[12] DeLuca JM, Kearney MH, Norton SA, Arnold GL (2011). Parents’ experiences of expanded newborn screening evaluations. Pediatrics.

[13] Gehtland LM, Paquin RS, Andrews SM, Lee AM, Gwaltney A, Duparc M (2022). Using a patient portal to increase enrollment in a newborn screening research study: observational study. JMIR Pediatr Parent.

[14] Abad PJ, Sibulo MS, Sur AL (2019). Role of the nurse in newborn screening: Integrating genetics in nursing education and practice. Philipp J Nurs.

[15] Mak CM, Law EC, Lee HH, Siu WK, Chow KM, Au Yeung SK (2018). The first pilot study of expanded newborn screening for inborn errors of metabolism and survey of related knowledge and opinions of health care professionals in Hong Kong. Hong Kong Med J.

[16] Moody L, Atkinson L, Kehal I, Bonham JR (2017). Healthcare professionals’ and parents’ experiences of the confirmatory testing period: a qualitative study of the UK expanded newborn screening pilot. BMC Pediatr.

[17] Newcomb P, True B, Wells JN, Walsh J, Pehl S (2019). Informing new mothers about newborn screening bloodspot repositories during postpartum hospitalization. MCN Am J Matern Child Nurs.

[18] Park SH, Woo MS, June KJ, Yu JO (2020). Analysis of women’s concern about pregnancy and child birth in the internet community. Perspect Nurs Sci.

[19] You MA, Baek EB, Kang NG (2021). The influences of mother’s eHealth literacy, health information orientation, and social support on the use of internet on health promotion behaviors for their children. J Health Info Stat.

[20] Moon RY, Mathews A, Oden R, Carlin R (2019). Mothers’ perceptions of the internet and social media as sources of parenting and health information: qualitative study. J Med Internet Res.

[21] Yu JO, June KJ, Park SH, Woo MS (2020). Content analysis of mothers’ questions related to parenting young children in internet parenting community. J Korean Soc Matern Child Health.

[22] Blei DM (2012). Probabilistic topic models. Communications ACM.

[23] Lee J, Kim Y, Kwak E, Park S (2021). A study on research trends for gestational diabetes mellitus and breastfeeding: Focusing on text network analysis and topic modeling. J Korean Acad Soc Nurs Educ.

[26] Jeong SJ (2020). A topic modeling approach to the analysis of domestic environmental education research trend: focusing on Journal of Korean Society for Environmental Education. Korean J Environ Educ.

[27] Lee SS (2016). A study on the application of topic modeling for the book report text. J Korean Libr Info Sci Soc.

[28] Naili M, Chaibi AH, Ghézala HB (2017). Arabic topic identification based on empirical studies of topic models. ARIMA Journal.

[30] Hong KB, Park JY, Chang YP, Yu J (2009). Thyroid dysfunction in premature infants. Korean J Pediatr.

[31] Conway M, Vuong TT, Hart K, Rohrwasser A, Eilbeck K (2022). Pain points in parents' interactions with newborn screening systems: a qualitative study. BMC Pediatr.

[32] Park JH, Moon CJ, Jung MH, Sung IK, Kim SY (2016). Perinatal factors associated with the preterm thyroid screening test. Korean J Perinatol.

[33] Yoon HR, Ahn S, Lee H (2019). Evaluation of the congenital hypothyroidism for newborn Screening program in Korea: a 14-year retrospective cohort study. J Korean Soc Inherit Metab Dis.

[34] de Miranda KS, dos Santos IC, de Almeida Neto OP, Calegari T (2020). Barriers experienced by nurses in newborn screening: a integrative review. Revista de Atenção à Saúde,.

[35] Timmermans S, Buchbinder M (2010). Patients-in-waiting: living between sickness and health in the genomics era. J Health Soc Behav.

[36] Kamleh M, Williamson JM, Casas K, Mohamed M (2021). Reduction in newborn screening false positive results following a new collection protocol: a quality improvement project. J Pediatr Pharmacol Ther.

[37] Rosettenstein KR, Lain SJ, Wormleaton N, Jack MM (2021). A systematic review of the outcomes of false-positive results on newborn screening for congenital hypothyroidism. Clin Endocrinol (Oxf).

[38] IJzebrink A, van Dijk T, Franková V, Loeber G, Kožich V, Henneman L (2021). Informing parents about newborn screening: a European comparison study. Int J Neonatal Screen.

[40] Barr JA, Tsai LP, Welch A, Faradz SM, Lane-Krebs K, Howie V (2018). Current practice for genetic counselling by nurses: an integrative review. Int J Nurs Pract.

[41] Choi KS, Kim HJ, Jang ES, Park JA (2010). A study of the curriculum of genetics nursing education. J Korean Oncol Nurs.

[42] Choi H (2014). Undergraduate nursing students’ perceived knowledge and attitudes toward genetics and nursing competencies for genetics. J Korean Biol Nurs Sci.

[43] Sohn YB (2015). A diagnostic algorithm of newborn screening for galactosemia. J Korean Soc Inherit Metab Dis.

